# Concurrent visual and acoustic tracking of passive and active delivery of nanobubbles to tumors

**DOI:** 10.7150/thno.51316

**Published:** 2020-09-23

**Authors:** Carly Pellow, Eric C. Abenojar, Agata A. Exner, Gang Zheng, David E. Goertz

**Affiliations:** 1 Department of Medical Biophysics, University of Toronto, Toronto, Ontario, Canada.; 2 Princess Margaret Cancer Research Centre, Toronto, Ontario, Canada.; 3 Sunnybrook Research Institute, Toronto, Ontario, Canada.; 4 Department of Radiology, Case Western Reserve University, Cleveland OH 44106, United States

**Keywords:** extravasation, intravital imaging, multiphoton microscopy, nanobubble, ultrasound

## Abstract

**Background:** There has been growing interest in nanobubbles for their potential to extend bubble-mediated ultrasound approaches beyond that of their larger microbubble counterparts. In particular, the smaller scale of nanobubbles may enable them to access the tumor extravascular compartment for imaging and therapy in closer proximity to cancer cells. Compelling preliminary demonstrations of the imaging and therapeutic abilities of nanobubbles have thus emerged, with emphasis on their ability to extravasate. However, studies to date rely on indirect histologic evidence that cannot confirm whether the structures remain intact beyond the vasculature - leaving their extravascular potential largely untapped.

**Methods:** Nanobubble acoustic scattering was assessed using a recently reported ultra-stable formulation at low concentration (10^6^ mL^-1^) and frequency (1 MHz), over a range of pressures (100-1500 kPa) in a channel phantom. The pressure-dependent response was utilized as a basis for *in vivo* experiments where ultrasound transmitters and receivers were integrated into a window chamber for simultaneous intravital multiphoton microscopy and acoustic monitoring in tumor-affected microcirculation. Microscopy and acoustic data were utilized to assess passive and active delivery of nanobubbles and determine whether they remained intact beyond the vasculature.

**Results:** Nanobubbles exhibit pressure-dependent nonlinear acoustic scattering. Nanobubbles are also found to have prolonged acoustic vascular pharmacokinetics, and passively extravasate intact into tumors. Ultrasound stimulation of nanobubbles is shown to actively enhance the delivery of both intact nanobubbles and shell material, increasing their spatial bioavailability deeper into the extravascular space. A range of acute vascular effects were also observed.

**Conclusion:** This study presents the first direct evidence that nanobubbles passively and actively extravasate intact in tumor tissue, and is the first to directly capture acute vascular events from ultrasound-stimulation of nanobubbles. The insights gained here demonstrate an important step towards unlocking the potential of nanobubbles and extending ultrasound-based applications.

## Introduction

Microbubbles have been in clinical use for decades as biomedical ultrasound contrast agents, with global approval for echocardiography and sanctioning in numerous countries for breast, kidney, liver, spleen, pancreas, gastrointestinal, and urogenital imaging 1, 2. Current formulations are comprised primarily of bubbles approximately 1-10 µm in diameter, with thin compliant shells encapsulating a high molecular weight gas core 3. Upon systemic injection, microbubbles circulate within the vasculature and oscillate in response to ultrasound resulting in distinct acoustic signatures which are exploited for imaging contrast 4. For example, at sufficiently high pressures, the bubbles' volumetric oscillations scatter energy coupled into frequency bands at integer multiples, or harmonics, of the driving frequency. Beyond imaging, sufficiently large oscillations can induce an array of therapeutically relevant biological effects, ranging from transiently increasing microvascular permeability to sustained microvascular damage 5.

Recently, there has been growing interest in submicron ultrasound cavitation agents to extend ultrasound-mediated applications. Submicron agents are of a size that permits higher number densities which can present advantages for high frequency ultrasound imaging, where the focal volume is reduced relative to conventional diagnostic frequencies 6. Little work has been done from a therapeutic perspective, though initial evidence indicates that submicron formulations can permeabilize the blood-brain-barrier 7, 8 and enhance drug delivery to tumors 9-15. Importantly, if the agents are sufficiently small they can further gain access to the extravascular compartment of tumors 16, which is accompanied by a host of advantages. From an imaging perspective, extravasated nanobubbles (NBs) can potentially enable detection of leakage associated with disease processes as well as facilitate detection of cell-surface markers in tissue. From a therapeutic standpoint, extravascular NBs can be stimulated by ultrasound for cavitation-based therapy in closer proximity to target cells and presents an opportunity for sonodynamic therapy. Further, nanoparticles exhibit structural versatility, improved pharmacokinetics, and multifunctionality. Shrinking conventional ultrasound contrast agents to the nanoscale consolidates with them the abilities of traditional nanoparticles, bringing new possibilities 16. Broadly, this class of submicron ultrasound agents 16 includes echogenic liposomes 17-19, polymersomes 20, gas vesicles 21-23, cavitation seeds 14, 24, nanodroplets 15, 25, 26, and nanobubbles 27-29.

Here we focus on NBs, consisting of encapsulated bubbles on the order of hundreds of nanometers. NBs have been shown to be present in large quantities in clinical microbubble formulations 30, though their potential has been largely untapped. Numerous reports have begun to emerge for the formulation of stable NBs 31-35 with applications of conventional ultrasound contrast imaging 34-41, targeted imaging 42-44, and as predictive markers 40, 45. Several reports have further demonstrated compelling therapeutic evidence of targeted 9-11, 46-48 and loaded 8-13, 46-49 NBs with both passive and active (ultrasound-triggered) approaches.

Despite this growing body of research, works employ ultrasound parameters with little or no basis on NB acoustic scattering, and - though intact extravasation is crucial to many future applications - rely on indirect histologic or macro-scale ultrasound imaging evidence. In this work we employ a fluorescently tagged version of a recently reported ultra-stable NB formulation 34. We first investigate the scattering response of NBs in a vessel phantom and demonstrate their ability to exhibit distinct nonlinear activity that persists. We then employ a custom dorsal window chamber apparatus to achieve simultaneous intravital multiphoton microscopy optical and acoustic monitoring 50 of NBs in tumor-affected vasculature. Using phantom scattering insights as a rational basis for parameter selection, visual observations are complemented by acoustic pharmacokinetic analysis to demonstrate that these NBs display prolonged stability, circulating in the vasculature for 20 minutes prior to dissolution. We then provide direct confirmation of shell material and intact NBs in the extravascular space after passive intravenous injection. To assess the ability of NBs to actively enhance microvascular permeability, ultrasound is then applied during NB injection and the resultant spatiotemporal extravasation profile and acute vascular effects are assessed. It is demonstrated that ultrasound-stimulated NBs extravasate intact with greater spatial bioavailability for extended imaging and therapeutic applications beyond the vasculature.

## Materials and Methods

### Agent preparation

Texas Red-labeled NBs were formulated using a modified version of a previously reported technique (developed and characterized *in vitro* and *in vivo* with a clinical scanner in 34). To prepare the base lipid solution, 144 µL of N-(Texas Red sulfonyl)-1,2-dihexadecanoyl-*sn*-glycero-3-phosphoethanolamine, triethylammonium salt (dissolved in chloroform to a concentration of 1 mg/mL; Texas Red® DHPE; Biotium, Inc.) was first added to a glass vial. The chloroform was then evaporated off in a hot water bath. Next, 6.1 mg of 1,2-dibehenoyl-*sn*-glycero-3-phosphocholine (DBPC C22; Avanti Polar Lipids Inc.), 2 mg of 1,2-dipalmitoyl-*sn*-glycero-3-phosphoethanolamine (DPPE, Corden Pharma), 1 mg of 1,2-dipalmitoyl-*sn*-glycero-3-phosphate (DPPA; Corden Pharma), and 1 mg of 1,2-distearoyl-*sn*-glycero-3-phosphoethanolamine-*N*-[methoxy(polyethylene glycol)-2000] (DSPE-mPEG2k; Laysan Lipids) were added to the glass vial and dissolved into 0.1 mL of propylene glycol by heating at 80 °C and sonicating. A mixture of 0.1 mL glycerol and 0.8 mL phosphate buffer solution heated to 80 °C was then added, and the resulting lipid solution was sonicated for 10 min at room temperature. The solution was then transferred to a 3 mL glass vial, capped with a rubber septum and aluminum seal, and sealed with a vial crimper. Air was removed from each vial with a 30 mL syringe, and replaced with octafluoropropane gas (C_3_F_8_, Electronic Fluorocarbons). Samples were activated by high shear gas dispersion in a VialMix (Bristol-Myers Squibb Medical Imaging Inc.) for 45 s. NBs were then isolated by differential centrifugation at 50 g for 5 min with the vial inverted, and 500 µL of NB solution was withdrawn at 5 mm from the bottom with a 21 G needle. Excess Texas Red® DHPE dye was removed by passing the isolated NB solution through a Sephadex PD-10 column (Cytiva). NBs were then transferred to new vials filled with octafluoropropane gas, capped, and sealed. As in a previously explored storage technique, sample vials were frozen and stored at -80 °C until use 51.

**Figure 1:**
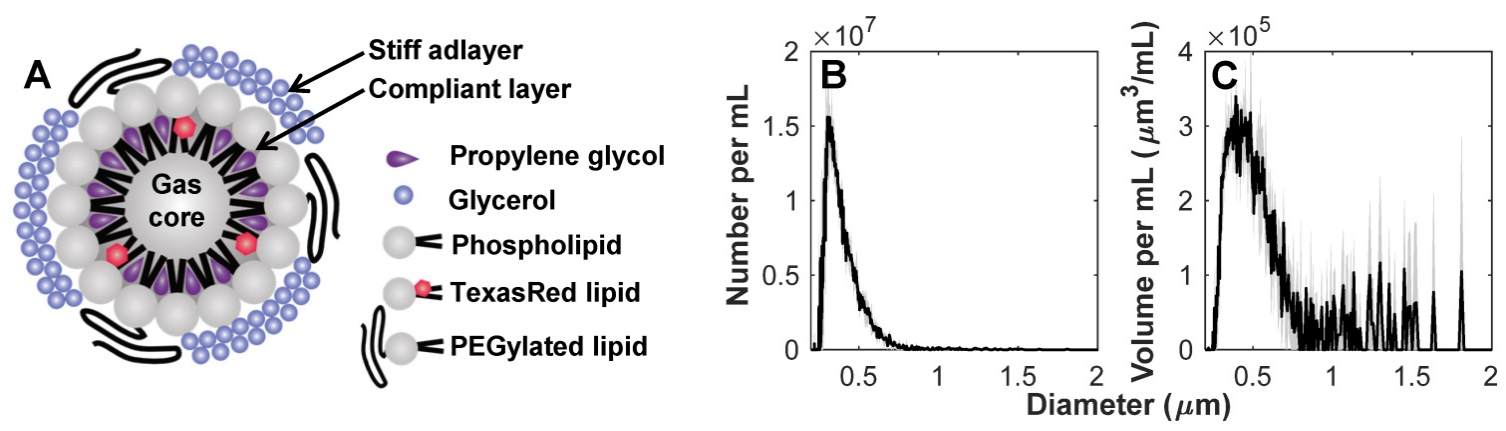
** Characterization of nanobubbles. (A)** Concept sketch of nanobubbles stabilized by a stiff adlayer of glycerol and compliant layer of propylene glycol. The agent was characterized with a Multisizer 4e (10 µm aperture) to have **(B)** number and **(C)** volume distribution modes of 0.303 and 0.392 µm, with a concentration of 8x10^8^ mL^-1^. Mean values (n = 3) are displayed with standard deviations in gray shading.

Prior to experiments, sample vials were thawed at room temperature. Agent was gently mixed, inverted, and 0.3 mL was extracted with a blunt 18 G needle into 0.7 mL saline. The diluted sample was passed gently through a 0.8 µm pore size syringe filter (Millipore Millex-GV) to isolate the NB population for use within 30 min after thawing. The agent size distribution was measured with a 10 µm aperture Multisizer 4e (Beckman Coulter Inc.) after thawing, filtration and dilution (**Figure 1**) to have a concentration of 8x10^8^ mL^-1^ and number and volume distribution modes of 0.303 and 0.392 µm, respectively (n = 3 vials).

### Benchtop acoustic scattering studies

**Figure 2:**
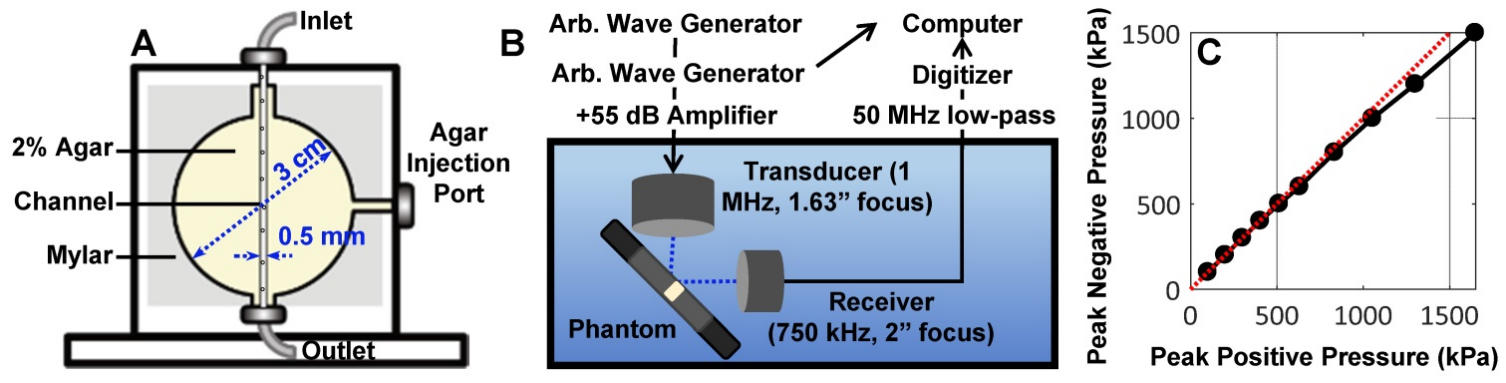
** Overview of the benchtop acoustic scattering studies. (A)** Schematic of channel phantom (0.5 mm diameter) intended to mimic a large vessel surrounded by tissue (2% agar gel). **(B)** Experimental configuration for passive cavitation detection of ultrasound-stimulated NBs in the phantom.** (C)** Hydrophone measurements of peak negative versus peak positive pressure at the transducer focus. These calibrations were utilized to assess NB scattering in the phantom as a function of pressure.

NB acoustic scattering was assessed at a low concentration of 10^6^ mL^-1^ in a vessel phantom. The phantom consisted of a 0.5 mm diameter channel cast with a needle in an acrylic chamber (3 cm x 3 cm circular cross-section; 1 cm length; 5 mm from the front face) filled with 2 % agar gel, held by mylar sheets (**Figure 2**A). Upon gelation, the needle was removed to create a channel, and the mylar was removed from the front face to minimize reflections. The phantom was placed in a degassed water bath such that each transducer (two were utilized for passive cavitation detection) focus (co-aligned) was situated 5 mm behind the phantom front interface and at the front of the channel, with the transducers at a 90° angle to each other and 45° to the phantom (**Figure 2**B). Agent was held stationary in the channel and replenished for subsequent acquisitions.

Two arbitrary waveform generators (Tektronix AFG3022B) were used to drive the transmit transducer (1MHz, 1” diameter, 1.63” focal length; C302-SU, Olympus NDT, USA). One of these was used to control pulsing parameters, while the second one was used to trigger pulses of set parameters for a set number of repetitions. The transmit transducer was driven at varied peak negative pressures (100 - 1000 kPa; calibrated with a 200 µm aperture hydrophone as in **Figure 2**C; HGL-0200, Onda Corporation, USA) for a pulse duration of 100 µs at a pulse repetition period of 1 ms repeated 100 times (n = 4 interrogations per pressure). Pulses were amplified by a 55 dB linear power amplifier (A150, E&I Inc, USA) prior to being sent to the transmit transducer. Agent scattering in the phantom was then passively detected with a broadband piezocomposite 750 kHz focused receive transducer (1” diameter, 2” focal length; IL0758HP, Valpey-Fisher, USA), filtered (50 MHz low-pass filter; Minicircuits, USA), and digitized at 125 MHz with a 14-bit PC-based oscilloscope (PicoScope 6402C, Pico Technology Ltd).

### Tumor cell line and animal preparation

**Figure 3:**
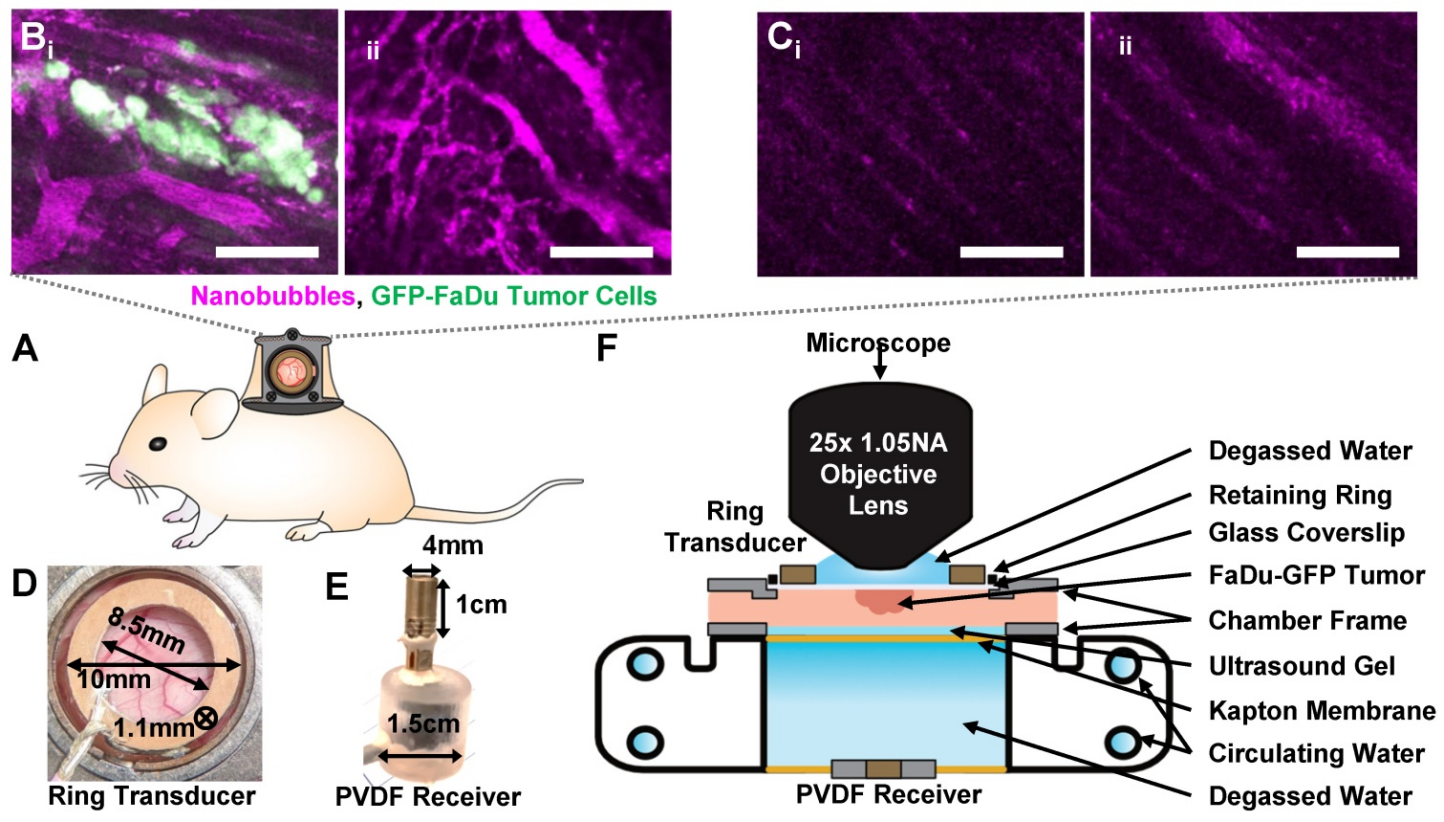
** Overview schematic of the integrated acoustic and dorsal window chamber microscopy setup. (A)** Side-view sketch of a mouse with a dorsal window chamber implanted, with a ring transducer and coverslip held in the window with a retaining ring. **(B)** Images of tumor tissue with dense tumor cell beds and noticeable vessel tortuosity. **(C)** Images of healthy tissue, with aligned, regular, straight vessels. Scale bar = 100 µm. **(D)** Photograph of the ring transducer held in the window chamber enabling acoustic stimulation during imaging, with dimensions.** (E)** Photograph of the acoustic receiver for detecting acoustic emissions behind the dorsal chamber, with dimensions.** (F)** Schematic of the multiphoton microscope interfacing with the dorsal window chamber and ultrasound transmit and receive transducers.

Green fluorescent protein (GFP) tagged human FaDu squamous cell carcinoma cells (AntiCancer Inc.) were cultured in 5% CO_2_/95% air at 37 °C. Cells were propagated in RPMI medium 1640 with L-glutamine (MultiCell Technologies Inc.), supplemented with 10% FBS, 100 U/mL penicillin, and 100 µg/mL streptomycin, and were trypsinized and harvested prior to confluency. All animal procedures were approved and conducted in compliance with the Animal Care Committee guidelines at Sunnybrook Research Institute, Canada. Six- to eight-week old BALB/c nude mice (Charles River) underwent dorsal skinfold window chamber implantation as in 50, 52. This technique involved the surgical implantation of a titanium frame to support the dorsal skinfold within a transparent window (**Figure 3**A). The upper layer of the dorsal skin was removed, and 2x10^6^ tumor cells in 30 µL of media were injected with a 30 G needle into the fascia in the window center, and a cover slip was placed over the opening (n = 15 mice). For inclusion in the study, mice with injected tumor cells were required to have both large visual regions of tumor cells within the volume-of-view, as well as noticeably affected (tortuous and redundant) vasculature (**Figure 3**B). Healthy mice were also utilized as vascular controls, where the dorsal chamber was implanted but no cells were injected (n = 5 mice), with noticeably straighter and less dense vasculature (**Figure 3**C). Studies were performed 8-10 days later.

For experiments, mice were anesthetized with isofluorane, and the tail vein was cannulated with a 27 G catheter. Mice were then placed on a heating pad on a removable microscope stage to maintain a core body temperature of 37 °C with feedback from a rectal thermistor (TC-1000; CWE Inc.). The window chamber coverslip was removed, the underlying exposed skinfold was wet with degassed saline, and a new 12 mm diameter, 150 µm thick coverslip with an in-house lead zirconate titanate (PZT-4) ring transducer fixed with cyanoacrylate adhesive to the top surface was placed overtop the exposed skin and held in the chamber with an internal retaining ring (**Figure 3**D). The underside of the window chamber was coupled by ultrasound gel to a degassed water bath reservoir heated with a circulating water heater (T/Pump Model TP-500; Gaymar) to maintain the dorsal skinfold temperature at 37 °C. On the bottom of the reservoir was an in-house polyvinylidene difluoride (PVDF) receive transducer for passive acoustic monitoring (**Figure 3**E). The stage was then transferred to the multiphoton microscope for the study.

### Multiphoton microscope settings

A water immersion 25x 1.05 NA objective lens with a field-of-view (FOV) of 509 µm x 509 µm (XLPN 25x, NA 1.05; Olympus) was positioned over the dorsal window chamber. Laser scanning was performed at 900 nm with a multiphoton microscope (FV1000MPE; Olympus) and a mode-locked Titanium Sapphire tunable laser (690-1040 nm; MaiTai Spectra-Physics). Fluorescent emissions following bandpass filtering were collected by a photomultiplier tube following bandpass filtering of 410-460 nm for collagen and 495-540 nm for FaDu-GFP, or by gallium arsenide phosphide (GaAsP) detectors following bandpass filtering of 575-645 nm for Texas Red tagged NBs.

### Ultrasound parameters

An in-house lead zirconate titanate (PZT-4) ring transducer 53 was matched to a 50 Ω impedance, 0° phase load with a custom matching circuit, with a driving frequency of 1.13±0.07 MHz in thickness mode. The ring transducer was calibrated with a 75 µm aperture hydrophone (model NH0075, Precision Acoustics) in a degassed water bath with the same configuration as the experiment (i.e. air-backed with a droplet of degassed water in the inner part of the cylinder, coupled with a water-immersion lens and held within a titanium frame).

For sonication, the ring transducer was air-backed, with a droplet of degassed water in the inner part of the cylinder for compatibility with the water-immersion lens. Two arbitrary waveform generators (Tektronix AFG3022B) were used to drive the ring transducer. One of these was used to control pulsing parameters, while the second one was used to trigger pulses of set parameters for a set number of repetitions. Three different pulsing schemes were utilized. 'Probe' pulses were employed to assess the acoustic vascular kinetics of the NBs. Probes were sent at the ring transducer fundamental frequency at 300 kPa peak negative pressure with a pulse length of 100 µs, pulse repetition period (PRP) of 4 s to allow vascular replenishment, 5 times. 'Destructive probe' pulses were used to assess whether NBs extravasated intact. Destructive probes were sent at 1 MPa peak negative pressure with a pulse length of 100 µs, PRP of 10 ms, 50 times. After the first hit, a 10 s waiting period was utilized to enable possible vascular replenishment, after which a second hit was transmitted. 'Sonication' pulses were intended to enhance vascular permeability to increase delivery. Sonication pulses were sent at 500 kPa peak negative pressure 10 s after agent injection with a pulse length of 2.5 ms, a PRP of 4 s to allow for vascular replenishment, to 2 min (30 bursts).

Pulses were first attenuated by 20 dB, amplified with a 53 dB RF power amplifier (E&I Ltd.), filtered by a 3 MHz low-pass filter, and transmitted through the matching circuit to the ring transducer. Passive cavitation detection was achieved with an in-house broadband polyvinylidene fluoride (PVDF) receiver centred at 10 MHz. Receive signals were digitized at 125 MHz with a 14-bit PC-based oscilloscope (PicoScope 6402C, Pico Technology Ltd).

### In vivo experimental procedure

As in 52, 50, the objective lens and ring transducer were colocalized and imaging was completed near the surface of the dorsal skinfold (up to a depth of 150 µm) to both maintain a high signal-to-noise ratio for the microscope and remain close to the transducer acoustic focus (with a lateral FWHM of ~500 µm). Prior to agent injections and laser scanning, baseline cavitation data was acquired with the same pulse sequences as would be utilized later. An injection of 0.1 mL TR-NBs at a concentration of 8x10^8^ mL^-1^ followed by a 0.08 mL saline flush was then administered via the tail vein catheter, and a baseline XYZ volume stack was acquired to create a 3D tumor vascular map. Volume stacks were acquired with 512x512 pixels (509.12 µm x 509.12 µm, resolution of 0.9944 µm/pixel) to a Z-depth of 0-150 µm in 1 µm increments at 2 µs/pixel. A second injection was performed 30 min after baseline acquisitions, marking the start time (*t*=0) of the experiments.

**Figure 4:**
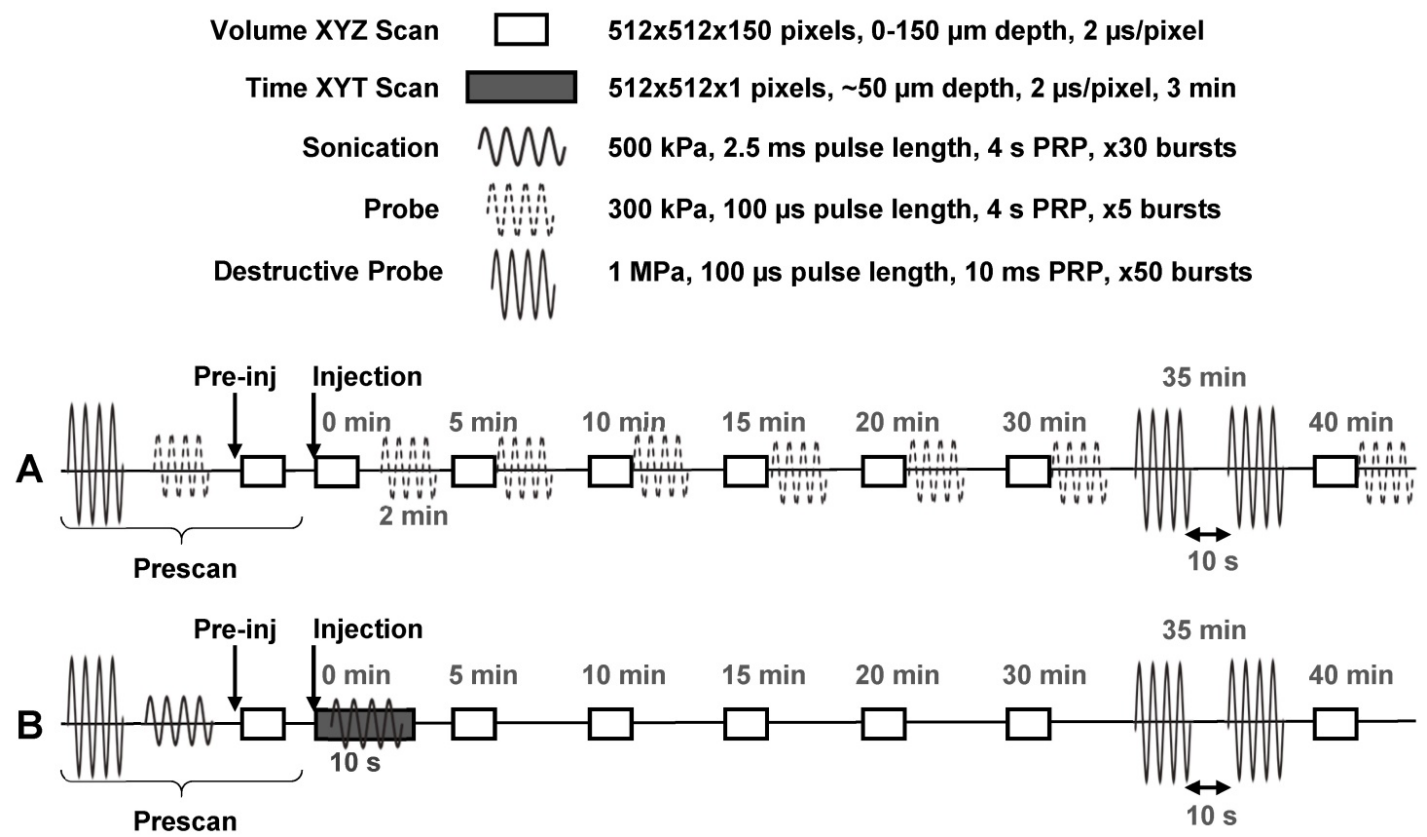
** In vivo experimental timing diagram. (A)** Scheme utilized to determine the vascular acoustic pharmacokinetic profile of the NBs, and test whether NBs extravasated intact (n = 10 mice with tumors, n = 5 healthy controls). **(B)** Scheme used to assess whether ultrasound stimulation of NBs upon injection can actively enhance vascular permeability to increase delivery; and whether there is a resultant increase in intact extravascular NBs (n = 5 mice with tumors, n = 2 healthy controls).

Two different experimental schemes were utilized (**Figure 4**): The first scheme (n = 10 mice) was intended to determine the vascular acoustic pharmacokinetic profile of the NBs, and to test whether NBs extravasated intact. In this scheme, probe pulses (300 kPa, 100 µs pulse length, 4 s PRP, 5x) were sent at 2, 5, 10, 15, 20, 30, and 40 min after injection. At 35 min, destructive probe pulses (1 MPa, 100 µs pulse length, PRP 10s, 50x; repeated after 10 s waiting period) were transmitted to acoustically determine if NBs extravasated intact. This scheme was also performed in healthy control mice (n = 5), to demonstrate that extravasation does not passively occur in the absence of leaky tumor vasculature. The second scheme (n = 5 mice) was utilized to assess whether ultrasound stimulation of NBs upon injection could actively enhance vascular permeability to increase delivery and determine whether there is a resultant increase in intact extravascular NBs. For this sequence, sonication pulses (500 kPa, 2.5 ms pulse length, 4 s PRP, 30x) were delivered 10 s after injection. These parameters are within range of those shown to give rise to drug delivery in conjunction with microbubbles.

During sonication, a time XYT stack (512x512 pixels, 2 µs/pixel; the same lateral and temporal resolution as the volume stacks) was acquired at a pre-selected tissue plane of depth between 50-100 µm such that tumor vessels of various sizes could be visualized with good SNR for 3 min. At 35 min, destructive probe pulses were transmitted to acoustically probe for the presence of intact extravascular NBs following active (ultrasound-mediated) delivery. This scheme was also performed in healthy control mice (n = 2), to demonstrate the extent of ultrasound-NB-mediated delivery in tumor compared to healthy vasculature. Throughout both experimental schemes, volume scans were acquired to visually monitor possible extravasation and vascular effects for up to 40 min following injection.

### Multiphoton microscopy data analysis

Multiphoton fluorescence images were analyzed in MATLAB with the assumption that fluorescence is proportional to concentration. The 575-645 nm channel (corresponding to NB signal) was corrected for GFP bleed-through, median filtered in 3-dimensions, and contrast enhanced via contrast-limited adaptive histogram equalization. A binarized 3D vessel mask from the baseline volume stack was created through iterative thresholding. Intra- and extra-vascular compartments were then segmented for spatiotemporal analysis by applying the mask and its inverse to the longitudinal images acquired, respectively. A Euclidean distance transform of the segmented 3D vascular mask was then performed to create a distance map from each extravascular pixel to the nearest vascular structure and applied to the extravascular compartment. Boundary effects were removed by truncating the volume by 40 pixels on all sides. Extravascular compartment fluorescence was normalized with respect to the compartment volume at each distance away from the nearest vessel, and to baseline fluorescence (time 0 min, distance 0-2 µm).

In the sonication group, triggered drug release over the time-course of ultrasound exposure was additionally examined, with the caveat that this could only be assessed within a pre-selected tissue plane depth. Here, the first 10 frames of the time XYT stack (5.18 s; prior to sonication) of the pre-selected tissue plane depth were utilized to create a 2D vascular mask. This mask was applied to the stack to assess the increase in extravascular compartment fluorescence over time normalized with respect to compartmental volume and baseline fluorescence (frames 1-10). All data is displayed as the mean and standard deviation unless otherwise indicated.

### Cavitation data analysis

Acoustic data was post-processed in MATLAB. Receive signals were digitally filtered (0.3 MHz high-pass and 5 MHz low-pass; 5th order bandpass Butterworth filter) and multiplied by a Hanning window of 50 µs length. For benchtop experiments, the Hanning window was centred on the received 100 µs signal. For *in vivo* experiments, the window was set to begin from the start of the received signal for the 2.5 ms sonication scheme; and was centered on the received signal for the 100 µs probe and destructive probe schemes. Windowed received signals were then zero-padded to a frequency resolution of 12.5 kHz per division prior to computing the Fourier transform and normalizing to the maximum received power in the first burst. For benchtop experiments, power spectra were then integrated over the fundamental frequency, 2nd harmonic, subharmonic, and inertial cavitation (between the fundamental and first ultraharmonic frequencies) bands with a bandwidth of -6 dB of the main lobe. For *in vivo* experiments, power spectra were integrated over the fundamental frequency and 2nd harmonic with a bandwidth of -6 dB of the main lobe. All data is displayed as the mean and standard deviation unless otherwise indicated. Significance was evaluated by a one-way analysis of variance (ANOVA) and a multiple comparison test.

## Results and Discussion

### Nanobubbles exhibit sustained nonlinear acoustic scattering

Despite the growing use of NBs, only a few studies have directly examined NB acoustic scattering systematically with a view to developing relevant ultrasound schemes 54, 55. In these works, scattering of dilute (10^6^ mL^-1^) suspensions of porphyrin-lipid NBs was assessed, and found to initiate nonlinear scattering in a pressure threshold-dependent matter at low (3-8 MHz) 54 and high 55 (12.5-30 MHz) frequencies. Another study assessed a higher concentration (10^9^ mL^-1^) of NBs incorporating propylene glycol and glycerol similar to the NBs in this study, and found contrast enhancement at 7-12 MHz over pressures ranging from 245-465 kPa 34.

In this work, NBs were formulated with a membrane of contrasting elastic properties 34. The compliant phospholipid encapsulating layer included propylene glycol as an edge activator for increased deformation 56-58, and glycerol as a membrane stiffener to increase buckling 59. The formulation was further doped with lipid-conjugated Texas Red for fluorescent tracking. The agent size distribution was determined with a 10 µm aperture Multisizer 4e (measuring 0.2 - 6 µm; Beckman Coulter Inc.) to be uncontaminated by larger particles which would dominate scattering, and to have number and volume distribution modes of 0.303 and 0.392 µm, respectively (n = 3; **Figure 1**).

**Figure 5:**
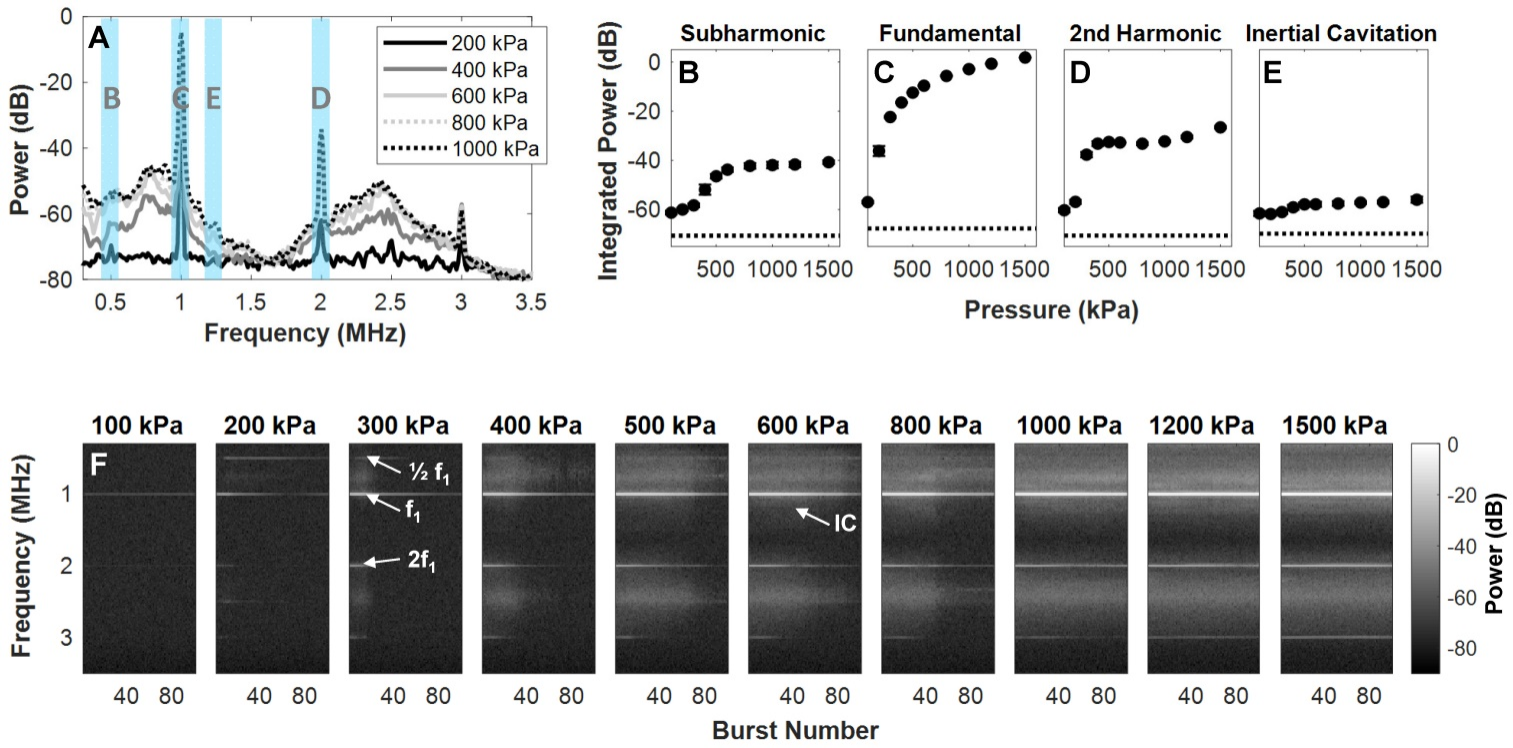
** Pressure-dependent scattered power from NBs. (A)** Representative received scattered power over a range of pressures. Highlighted regions denote frequency bands over which signals were integrated to calculate quantified scattering data in **(B)** subharmonic, **(C)** fundamental,** (D)** second harmonic, and **(E)** inertial cavitation regimes as a function of pressure. Data is averaged for the first 5 bursts of ultrasound exposure and over n = 4 interrogations per pressure. **(F)** NB power spectra as a function of successive bursts at different pressures (100-1500 kPa, in columns). Arrows denote the subharmonic (1/2 f_1_), fundamental frequency (f_1_), second harmonic (2f_1_), and inertial cavitation (IC) regimes.

NB scattering in response to ultrasound was assessed at a clinically relevant low concentration (10^6^ mL^-1^) in a vessel phantom (0.5 mm diameter). This low number density is aligned with current clinically acceptable microbubble doses based on gas volume 60, 61; the concentrations are therefore matched, but the NB dose here is of a lower gas volume (proportional to radius, *r*^3^) and surface area (proportional to *r*^2^). A calibrated custom setup (**Figure 2**) was utilized to determine behavior as a function of pressure (100-1500 kPa) with a driving frequency of 1 MHz, 100 µs pulse length, 1 ms pulse repetition period, for 100 successive bursts. Representative scattered power spectra are shown in **Figure 5**A. It should be noted that asymmetries arise due to nonlinear propagation at higher pressures (**Figure 2**C); at least a component of signals above ~800 kPa is due to nonlinear propagation as well as nonlinear scattering. The receiver is additionally a narrowband transducer with higher sensitivity at its fundamental frequency (750 kHz) and odd harmonics (2.25 MHz), thus inertial cavitation appears more prominent in these bands.

The fundamental and second and third harmonic responses are distinct at low pressures and increase with pressure. A subharmonic peak is also visible at and above 200 kPa, and broadband noise emerges at 400 kPa. A quantification of scattered signals as a function of pressure in the subharmonic, fundamental, second harmonic, and inertial cavitation regimes is shown in **Figure 5**B-E. At the fundamental frequency, power rises rapidly (100-500 kPa) and then more gradually (> 500 kPa) as a function of pressure. At the subharmonic and second harmonic, power begins to ascend quickly from 200-500 kPa, then begins to plateau. These plateaus occur with the emergence of broadband inertial cavitation above 400 kPa. It is thus evident that these NBs can exhibit nonlinear acoustic scattering at low concentrations and low-to-moderate pressures, and that they do so in a pressure-dependent manner that is similar to previous observations with other formulations 54, 55.

To assess signal persistence, scattering response as a function of successive pulses is shown in **Figure 5**F. At 200 kPa, the subharmonic and second harmonic are visible without the presence of broadband noise, though these harmonics are short-lived, decaying to noise levels after ~20 bursts at 200 and 300 kPa. These nonlinearities become more persistent at higher pressures with increasing inertial cavitation as a function of pressure. NBs are therefore further capable of sustained cavitation, as well as inertial cavitation for therapeutic avenues.

### Nanobubbles demonstrate extended acoustic vascular pharmacokinetics

Custom ultrasound transmitters and receivers were next integrated into a window chamber for simultaneous intravital optical and acoustic monitoring of intravenously injected NBs in tumor-affected microcirculation (**Figure 3**). First, NB acoustic vascular pharmacokinetics were assessed following passive infusion, in terms of their ability to initiate cavitation at a particular exposure level in plasma. The scheme utilized is outlined in **Figure 4**A: 100 µs, 300 kPa probes were sent in a short series (x5) with a 4 s PRP at 2, 5, 10, 15, 20, 30, and 40 min following injection. The 4 s PRP enables vascular replenishment of agent, while the short pulse length and moderate 300 kPa pressure enable reliably distinct nonlinear scattering without the presence of inertial cavitation, based on benchtop results (**Figure 5**). Thus, probing pulses briefly check for cavitation from intact NBs in the vasculature without causing destruction or permeabilization.

**Figure 6:**
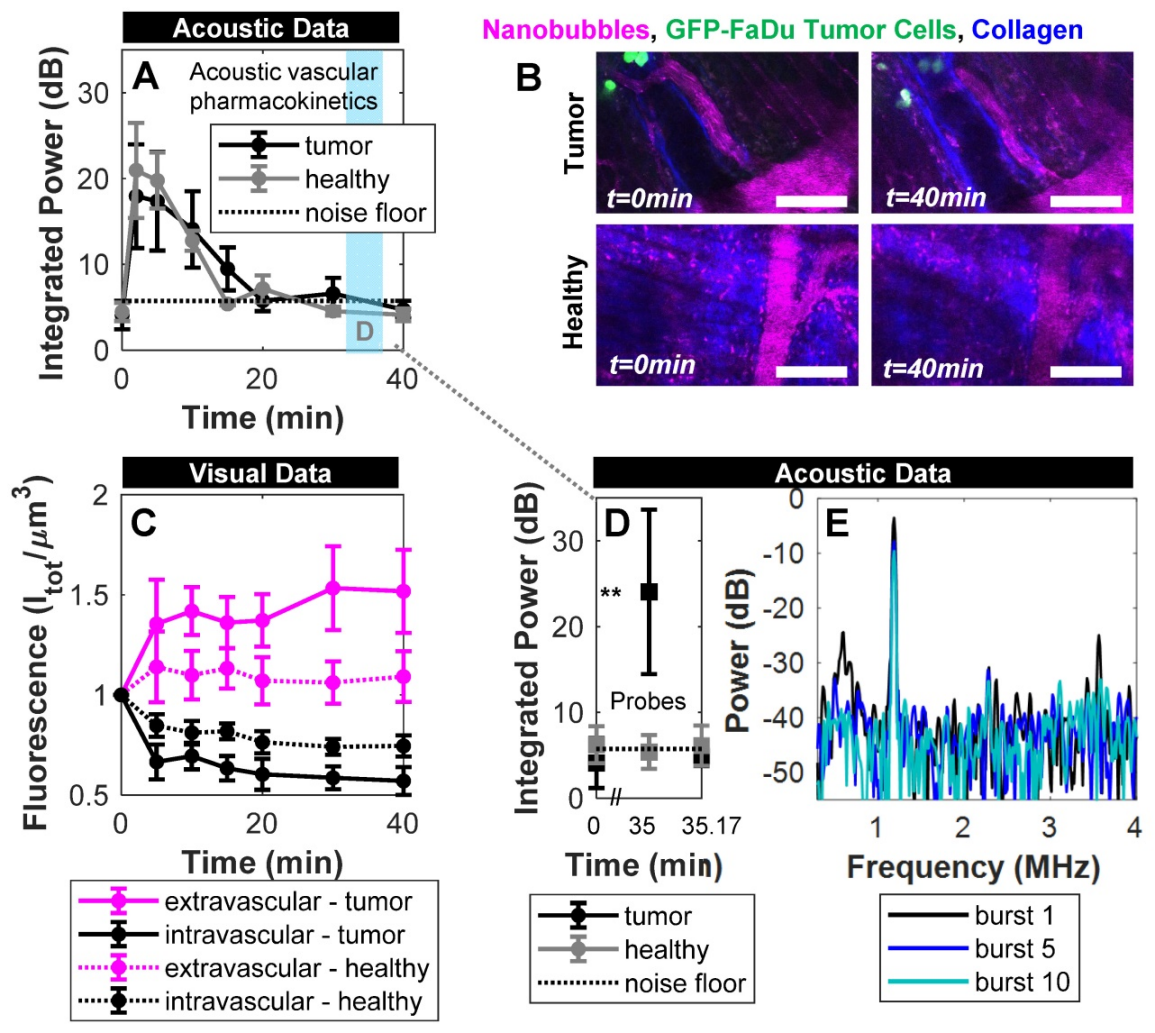
** Acoustic and visual tracking of NBs under passive delivery conditions. (A)** Acoustic vascular pharmacokinetic profile demonstrating the decay in acoustic integrated power as a function of time in tumor (n = 10) and healthy (n = 5) vasculature. Periodic probes (300 kPa, 100 µs, 4 s pulse repetition period, 5 bursts) detect NB cavitation *in vivo* for 20 min following injection. **(B)** Multiphoton images of NBs in tumor-affected and healthy vasculature over the course of passive delivery. A decrease in intravascular fluorescence and slight increase in extravascular fluorescence emerges slowly over 40 min, preferentially in tumor tissue. Scale bar = 100 µm. **(C)** Fluorescent tracking and vascular segmentation demonstrate preferential passive delivery of NBs to the extravascular compartment (volume of 509 µm x 509 µm x 150 µm) in tumors compared to healthy tissue. **(D)** Destructive probe pulse data (1000 kPa, 100 µs, 10 ms pulse repetition period, 50 bursts) intended to check for intact NBs in the extravascular compartment detected significant cavitation (** *p*<0.01) above baseline at 35 min in tumor vasculature, which is not detectable 10 s later due to destruction of stationary NBs. Conversely, destructive probe pulses did not detect cavitation above baseline in healthy mice. **(E)** Power spectra for the 1^st^, 5^th^, and 10^th^ burst in the first destructive probe sequence at 35 min. Elevated power at the fundamental frequency, second-, third-, and sub-harmonics are visible in the 1^st^ burst and decay with subsequent exposure.

**Figure 6**A shows the acoustic vascular pharmacokinetic profile of a low concentration NB bolus in tumor and healthy tissue. Prior to injection, probing pulses detected no cavitation (*t*=0 min). After injection, probes detected strong cavitation activity that decayed back to baseline by 20 min. This is far longer than typical microbubble formulations at equivalent doses that last on the order of 30 s to 5 min as measured with conventional imaging methods 62-64, with the caveat that the acoustic pharmacokinetic profile here was assessed with a different pulsing scheme. Other NB studies have compared NBs and microbubbles under the same exposure conditions, matching gas volume or number density, and found that NBs were more stable 7, 32, 33.

### Nanobubbles preferentially and passively extravasate intact into tumors

Following infusion, visual and acoustic data were utilized to determine whether NBs passively extravasate from tumor vasculature, and crucially, whether they extravasate intact. Works to date employ imaging of tumors or histology to demonstrate extravasation; however, the ultrasound imaging methods utilized did not have sufficient resolution to determine whether detected bubbles are truly outside of vessels, and histology simply depicts extravasated shell material.

Here, the scheme in **Figure 4**A provided visual monitoring of extravasation of material over time, with example images shown in **Figure 6**B. Fluorescence in the vascular and extravascular compartments of tumor and healthy tissue over time is quantified and shown in **Figure 6**C. Over a 40 min timespan from injection, there was preferential passive extravasation in tumors (up to 1.5x baseline) compared to healthy controls (1.15x). There was correspondingly a slightly greater decrease in intravascular fluorescence in tumor tissue. While visual intravital monitoring provides evidence of extravasation, it does so without distinguishing between intact NBs and shell fragments. While shell fragments can have relevance for imaging (if tagged) or therapy (if loaded appropriately), here we determine whether at least a subset of the visual data is associated with intact NBs for an extended range of applications.

To determine whether any NBs extravasated intact, destructive probes were applied at 35 min - after the lower amplitude imaging probes determined that NBs were no longer intact in the vasculature. Destructive probes consisted of two 'hits' with the same parameters; a short pulse length of 100 µs at high pressure (1 MPa) with a short pulse repetition period of 10 ms, 50 times. After the first hit a 10 s waiting period was utilized, after which a second hit was transmitted. The high pressure was selected to ensure broadband emissions (**Figure 5**) for detection of a potential lower number density, and because NBs in a confining tissue-like environment require higher pressures for cavitation 54. The long waiting period between the hits enables possible vascular replenishment if the NBs are present in blood; if the first hit exhibits cavitation that quickly decays and does not return in the second hit, it would indicate the presence of intact NBs disrupted by the hits (i.e. extravascular stationary signal) rather than being replenished (i.e. vascular signal).

In **Figure 6**D, the first destructive probe pulse at 35 min did not detect cavitation in healthy controls, indicating the absence of intact NBs. In the tumor group, however, significant cavitation above baseline was elicited, that was not detectable 10 s later. Specifically, the first destructive probe hit yielded strong elevated power at the fundamental frequency, second-, third-, and sub-harmonics that decayed with rapid successive hits (**Figure 6**E) and did not return 10 s later after possible replenishment. This indicates the presence of stationary, intact NBs in the extravascular space of tumors that were destroyed during the first hit. With the same reasoning, it has been shown that NBs can be generated *in situ* from ultrasound-stimulated microbubbles and be actively delivered intact into the extravascular compartment 50. This is the first study to demonstrate that NBs passively extravasate intact with contained gas in tumors. Maintaining gas content in the extravascular compartment is crucial to fully realize the potential of NBs: For imaging, extravasating NBs can facilitate detection of leakage due to disease processes (tumors, insulitis, *etc.*) and detect cell-surface markers in tissue. From a therapeutic perspective, extravascular NBs can initiate cavitation-based therapy approaches deeper in tumor tissue and in closer proximity to tumor cells.

### Ultrasound stimulation actively enhances delivery of intact nanobubbles

While we have demonstrated that NBs passively extravasate intact in a preclinical tumor model, the enhanced permeability and retention effect has been clinically disputed 65. Therefore, NBs were next stimulated with ultrasound (500 kPa, 2.5 ms pulse length, 4 s pulse repetition period, 2 min total duration) upon injection to actively enhance blood-tissue permeability. These parameters are within range of those shown to give rise to drug delivery in conjunction with microbubbles, and are the same as those used in the previous simultaneous intravital and acoustic monitoring study that investigated the generation and extravasation of intact nanobubbles from ultrasound-stimulated microbubbles 50. Here the scheme in **Figure 4**B provided monitoring both over the timescale of the sonication (at a pre-selected depth) and beyond over the entire FOV.

**Figure 7:**
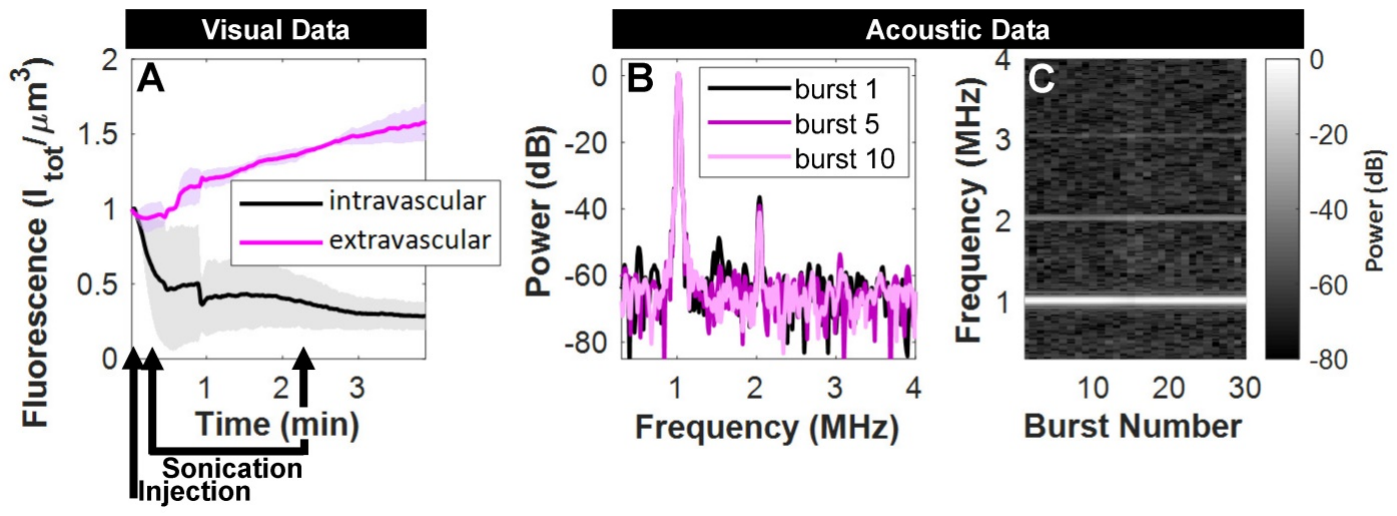
** Acoustic and visual monitoring during ultrasound-stimulation of NBs. (A)** Fluorescent tracking demonstrates a rapid rise in signal in the extravascular compartment upon sonication in mice with tumors. Visual data at this time resolution required pre-selection of a tissue plane, and is averaged here for the cases where vascular disruption was captured at this depth (n = 3/5). **(B)** Acoustic monitoring during sonication (500 kPa, 2.5 ms, 4 s pulse repetition period, 2 min total duration) demonstrates scattered power in the subharmonic and first ultraharmonic frequency bands in the first burst that is not visible thereafter, as well as distinct elevated fundamental and second harmonics that **(C)** persist over the duration of ultrasound-stimulation.

Over the timescale of the sonication (2 min), fluorescence in the extravascular compartment underwent a rapid rise to 1.5x its initial state in a pre-selected tissue plane in mice with tumors (**Figure 7**A). Acoustic monitoring during sonication (**Figure 7**B,C) denotes nonlinear scattering in the subharmonic and first ultraharmonic frequency bands that decay within a few bursts, as well as distinct elevated fundamental and second harmonics that persist. The lack of broadband noise implies that ultrasound-mediated active delivery was initiated under stable cavitation conditions. This is an important finding, as more violent inertial cavitation is often associated with hemorrhage and edema 66.

**Figure 8:**
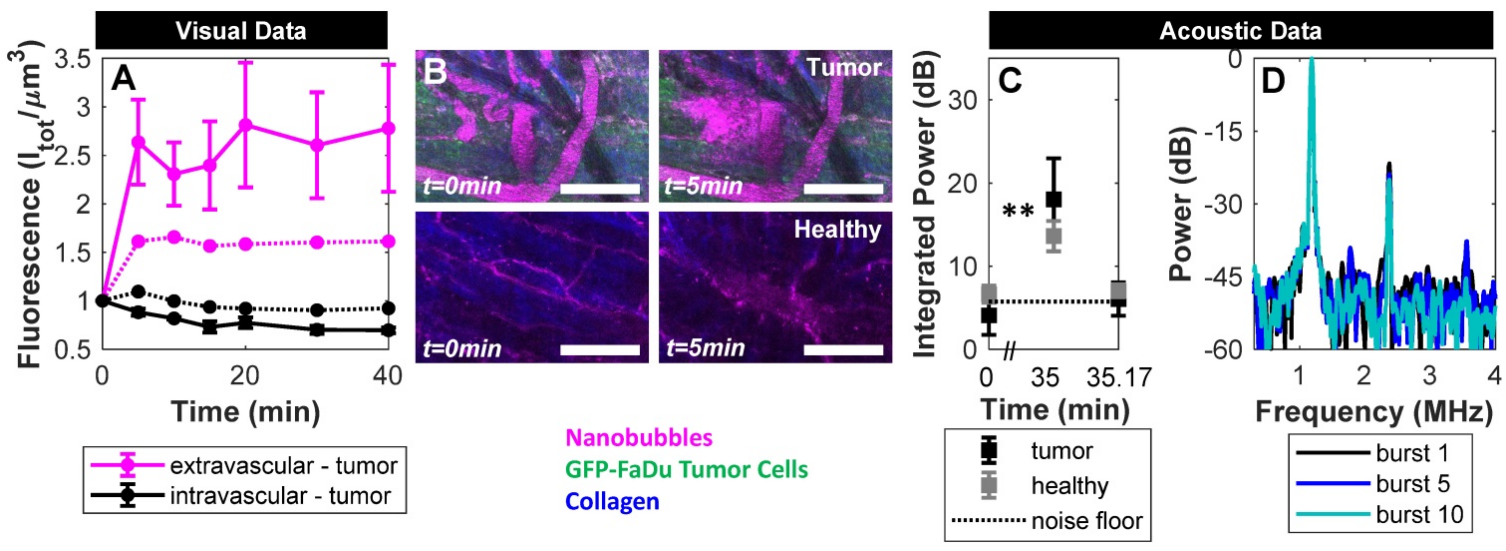
** Acoustic and visual tracking of NBs upon ultrasound-mediated active delivery. (A)** Fluorescent tracking illustrates a rapid rise in signal in the extravascular compartment during and immediately following sonication, followed by a plateau over the entire volume FOV. Sonication of NBs for 2 min beginning 10 s post-injection actively enhances extravasation in tumor (n = 5) and healthy (n = 2) groups, though to a greater extent in tumor tissue. **(B)** Representative images of ultrasound-mediated delivery in tumor and healthy tissue. Scale bar = 100 µm. **(C)** Destructive probe pulse data (1000 kPa, 100 µs, 10 ms pulse repetition period, 50 bursts) intended to check for intact NBs in the extravascular compartment detected significant cavitation above baseline at 35 min in both tumor (** *p*<0.01) and healthy groups following ultrasound-mediated active delivery. **(D)** Power spectra for the 1^st^, 5^th^, and 10^th^ burst in the first destructive probe sequence at 35 min following active delivery. Elevated power at the fundamental frequency, second-, third-, and first ultra-harmonics are visible in the 1^st^ burst and decay with subsequent exposure.

Over a longer timescale after sonication, extravascular fluorescence continued to increase to an average of 3x its initial state in mice with tumors, and to 1.6x its initial state in healthy mice (**Figure 8**A). Therefore, sonication of NBs for 2 min beginning 10 s post-injection actively enhances extravasation in both tumor and healthy groups, though to a greater extent in tumor tissue.

**Figure 9:**
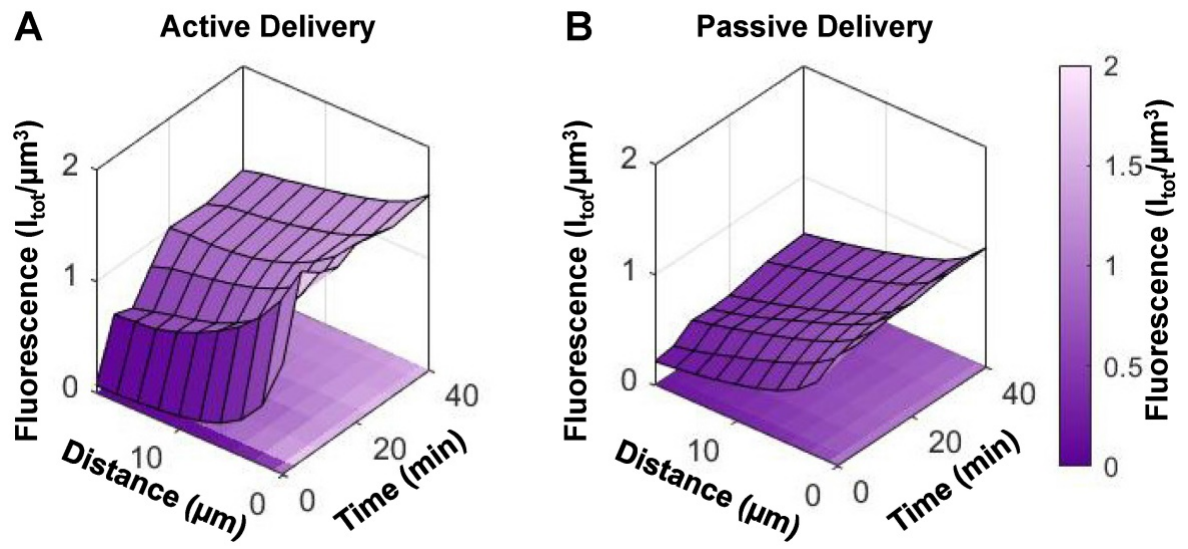
** (A)** Extravasation surface plot as a function of distance from the nearest vessel and time in mice with tumors that underwent active (ultrasound-mediated) delivery (mean of n = 5), and **(B)** passive delivery (mean of n = 10).

This visual data provides evidence of actively enhanced delivery of fluorescently labelled shell material which is of relevance for the use of drug-loaded NBs. However, this does not distinguish between shell fragments or intact NBs. To determine whether ultrasound-based active delivery enhanced intact NB delivery to the extravascular compartment, destructive probes (two 'hits' of 1 MPa, 100 µs pulse length, 10 ms pulse repetition period, 50 times; 10 s apart) were again utilized at 35 min. In **Figure 8**C, the first destructive probe pulse detected significant cavitation above baseline in tumor and healthy groups. This is indicative of the presence of intact NBs upon ultrasound-mediated active delivery. Cavitation levels in the active delivery groups (tumor-affected and healthy), however, were not significantly different, nor were levels comparing active and passive delivery to tumor-affected mice. Spatiotemporal extravasation was notably different between active and passive delivery to tumor-affected mice (**Figure 9**A, B), with ultrasound-mediated active delivery resulting in greatly enhanced extravasation distances.

While destructive probes do not provide spatial information on detected cavitation, aside from that inferred from behavior and time delays, it is possible that active delivery enhances intact NB penetration into tumors under the tested conditions: Indeed it has recently been shown that targeted NBs under ultrasound exposure are able to achieve deep penetration into rabbit clots, and with greater efficacy than stimulated MBs 67. Ultrasound stimulation of NBs has also been shown to enhance delivery and penetration of co-injected or loaded drugs in various preclinical tumor models 10, 11, 48, 49 and for blood-brain-barrier disruption 7, 8, though intact NB extravasation was not validated and microscale events were not visualized. A plethora of ultrasound parameters have been used, typically with frequencies between 1-10 MHz, durations on the order of a few minutes, but greatly varying acoustic pressures from 100 kPa to several MPa. The penetrating capacity of intact NBs warrants further investigation with a wider set of ultrasound parameters and a more advanced array receiver setup capable of more accurately localizing extravasated NBs. However, it should be noted that even with precise signal localization many factors will affect scattering. Scattered power will depend on NB concentration, proximity to other bubbles, NB diameter and nonlinear shell rheology, as well as microscopic local environment mechanical properties. Thus further exploration is warranted, yet will still require other concurrent monitoring methods (such as intravital multiphoton microscopy applied here).

### Ultrasound stimulation of nanobubbles elicits a range of biological effects

The integrated intravital visual and acoustic setup further provides powerful spatiotemporal insights. Such real-time monitoring of acute vascular effects resulting from ultrasound-stimulated bubble-based therapeutic approaches has only been utilized in a handful of studies in simplified models 68-70, in the absence of tumors 71-73, and without 52, 71-73 or with 50 recording acoustic response. However, these studies utilized microbubbles; no study to date has provided real-time intravital monitoring of acute vascular effects upon ultrasound stimulation of NBs. The events detailed below are further the first reported microscale observations of NB-vessel interactions in a complex tissue model (beyond glass catfish and the chorioallantoic membrane) outside of the brain.

**Figure 10:**
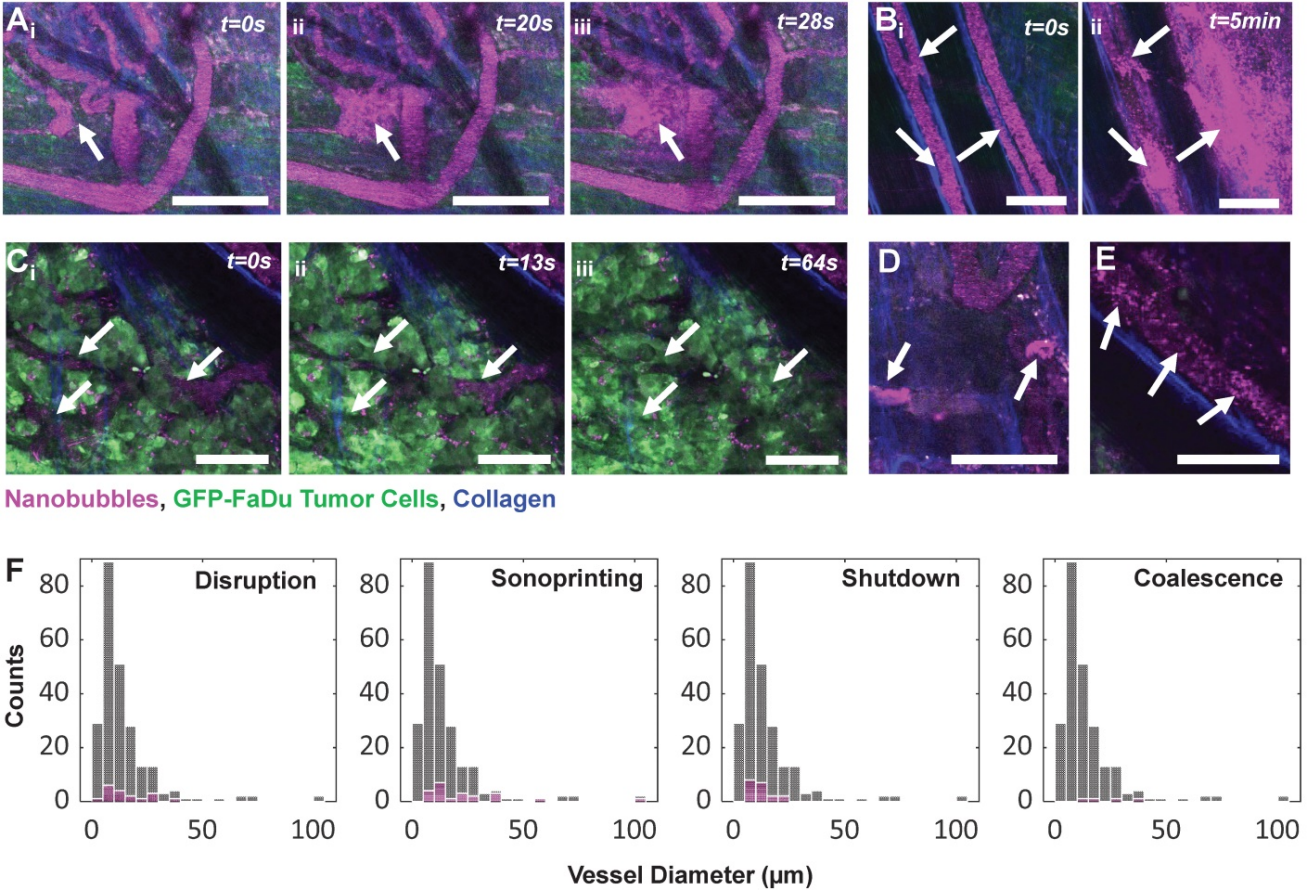
** NB-vessel interactions visualized with intravital multiphoton microscopy.** Fluorescent agent is colorized magenta, GFP-tagged FaDu tumor cells are green, and collagen is blue. **(A)** Rapid (within seconds) focal vascular disruption during ultrasound exposure (which begins at *t*=10 s). **(B)** Slower (within minutes), widespread vascular permeabilization occurring following ultrasound exposure. **(C)** Time-series depicting vascular shutdown in vessels within a dense tumor bed during sonication. **(D)** Stationary agent in vessels 25 min following injection and sonication, indicating uptake into or sonoprinting onto endothelial cells of the vessel wall. **(E)** Distinct moving clusters of agent signal 40 min following injection and exposure, hypothesized to be due to agent coalescence or attachment to red blood cells. Scale bar = 100 µm. **(F)** Histograms of vascular events (pink) organized by vessel diameter and event type, superimposed on total visualized vessels (gray).

While anecdotal, **Figure 10** depicts a range of captured NB-vessel interactions during or following ultrasound exposure. To provide context for the relative incidence of these events, we note that a range of 20-50 vessels were visible within the FOV of any given mouse. A total of n = 5 mice with tumor-affected vasculature were studied under ultrasound stimulation with 153 total vessels, and n = 2 healthy mice were assessed with 86 total vessels. None of the visualized events were observed in tumor or healthy mice in the absence of ultrasound exposure (n = 10 tumor, n = 5 healthy mice).

Two types of vascular disruption were visualized; rapid focal leakage (**Figure 10**A) which occurred during sonication, and slow widespread leakage (**Figure 10**B) occurring shortly following exposure. Microbubble-mediated vascular permeabilization for locally enhanced transport is one of the most widely studied therapeutic applications of ultrasound 5, 74, 75, and has advanced to clinical trials for transient blood-brain-barrier disruption 76 and enhanced delivery to tumors 77. Microbubble-mediated permeabilization can be achieved *via* thermal (hyperthermia) or mechanical (sonoporation, endocytosis) mechanisms 78. Microbubble oscillation-driven fluid flow (microstreaming, microjets) 79 and acoustic radiation forces 80 then assist in drug transport across the permeabilized vessel wall into tissue. Direct microscopic observations of permeabilization of individual vessels has been conducted in a variety of preclinical models for microbubbles 50, 68-73. This is the first study to visualize vascular permeabilization with ultrasound-stimulated nanobubbles, capturing disruption in 18 vessels (11/153 tumor vessels, 7/86 healthy vessels) with fluorescence of extravasated NBs colocalized with tumor cells.

Vascular shutdown was also observed (**Figure 10**C) in vessels within a dense tumor bed during sonication (19/153 tumor vessels, 0/86 healthy vessel). Antivascular therapy has been achieved *via* continuous wave ultrasound with high microbubble concentrations (resulting in macroscopic temperature elevations) 81, 82, as well as lower duty cycle pulsed ultrasound 83-86 with both high 84, 87 and low 83, 85, 88 microbubble doses. Antivascular therapy can inhibit tumor growth 85, 86, 89 and has further been reported to have strong synergistic effects when combined with radiotherapy 84 and anticancer agents 86, 88, 90. It is notable that the pressures and mechanical indices employed for the pulsed ultrasound work was higher than that in the present study. The role of bubble size on this approach has yet to be investigated, though these effects have been shown to be achievable with submicron bubbles 91.

In **Figure 10**D, agent was observed to either be taken up into or 'sonoprinted' onto endothelial cells of the vessel wall (observed in 22 vessels; 18/153 tumor vessels, 4/86 healthy vessels). This behavior has been reported with ultrasound-stimulated microbubbles using pressures above 300 kPa and short pulses 92, 93, as well as during stable cavitation with more moderate pressures (100-300 kPa) and longer pulses 50, 93, 94, and involves imprinting of shell material onto endothelial cells. This visual pattern notably remains the same before and after higher pressure destructive probe pulses, and results in similar cavitation levels regardless of incidence, and is therefore hypothesized to not contain gas. With this study being the first to employ intravital imaging of ultrasound-stimulated NBs, this is also the first report of sonoprinting with NBs.

In **Figure 10**E, 30-40 min after injection and exposure, agent appeared to either coalesce together, or alternatively attach to red blood cells in the vascular compartment. This was found to occur long after dissolution of gas from NBs in the vasculature and could contribute to the plateau in extravasation. This behavior was relatively rare, however (occurring in 4 tumor-affected vessels in 1 animal) and should be studied further.

While these biological effects were varied and phenomenological in nature, they all occurred under the same exposure conditions (500 kPa, 2.5 ms pulse length, 4 s pulse repetition period, 2 min total duration). The sonication parameters and injected bubble concentration were also utilized in a prior study of ultrasound stimulation of microbubbles to generate and facilitate the delivery of NBs to tumors 50. In the aforementioned work, extravascular fluorescence increased 5x, cavitation in accordance with stationary extravascular NBs was detected, and the spatial release profile was far greater than passive controls 50. Ultrasound stimulation for active delivery further occurred under stable cavitation conditions 50, as in the present study. In the current work, extravascular fluorescence increased by 3x under the same conditions with an equivalent number density of NBs (and therefore has a correspondingly lower gas volume and surface area doses). In both works, fluorescence was tracked *via* a tagged shell and thus corresponds to surface area of the bubbles, with 10 % fluorophore doping in the microbubble case, and 0.9 % doping in the NBs. With both cases occurring under stable cavitation and fewer vascular effects being observed in the present NB study, it is likely that a direct comparison of microbubbles and NBs with matched surface area content and fluorophore doping would result in greater delivery efficacy for NBs with fewer adverse events.

Microbubbles and NBs have been directly compared in a delivery context *in vivo* in only two works, both of which were for blood-brain-barrier opening: Bing *et al*. compared NBs to clinical microbubble formulations Optison® and Definity^TM^ under similar total gas volumes, with 0.5 MHz focused ultrasound and pressures ranging from 0.1 - 0.7 MPa with acoustic feedback control 7. It was found that NBs could achieve more reliable opening, though no histology was provided to compare resultant damage 7. Fan *et al.* compared NBs to the clinical microbubble formulation SonoVue®, with 1 and 10 MHz focused ultrasound and pressures ranging from 0.5 - 4.5 MPa 8. It was found that NBs achieved blood-brain-barrier opening with less hemorrhagic damage *via* histology, though it is unclear whether the two bubble groups were matched in terms of concentration or gas volume 8. Both of these studies were unable to monitor effects in real-time, highlighting the potential utility of the integrated optical and acoustic setup presented here for high temporal and spatial resolution observations of ultrasound-stimulated NBs.

## Conclusion

The present study has sought to gain insights into NBs and their potential for accessing the tumor extravascular space intact. NBs formulated with contrasting membrane elasticity architecture were found to exhibit sustained nonlinear acoustic scattering at clinically relevant low concentration (10^6^ mL^-1^) and frequency (1 MHz), over a range of pressures (100-1500 kPa). Simultaneous intravital optical and acoustic monitoring revealed that these NBs yield a lengthy acoustic vascular pharmacokinetic profile (20 min). It was then demonstrated that NBs preferentially and passively extravasate intact with contained gas into tumors (1.5x baseline) compared to healthy tissue (1.15x), and that ultrasound-stimulation further enhances their delivery (5x) and spatial bioavailability. Finally, ultrasound-stimulation of NBs was found to elicit a range of biological effects that either have not been previously reported or directly observed with NBs. These insights substantiate the immense potential that NBs can offer for extending ultrasound-based applications beyond the vascular compartment.

## Abbreviations

2D: two-dimensional
3D: three-dimensional
ANOVA: analysis of variance
DHPE: 1,2-dihexadecanoyl-*sn*-glycero-3-phosphoethanolamine
DBPC: 1,2-dibehenoyl-*sn*-glycero-3-phosphocholine
DPPE: 1,2-dipalmitoyl-*sn*-glycero-3-phosphoethanolamine
DPPA: 1,2-dipalmitoyl-*sn*-glycero-3-phosphate
DSPE-mPEG2k: 1,2-distearoyl-*sn*-glycero-3-phosphoethanolamine-*N*-[methoxy(polyethylene glycol)-2000]
FOV: field of view
GaAsP: gallium arsenide phosphide
GFP: green fluorescent protein
NB: nanobubble
PVDF: polyvinylidene difluoride
PZT-4: lead zirconate titanate
